# Anaesthesia Challenges in Patients With Chronic Kidney Disease: A Clinical Approach From Internal Medicine

**DOI:** 10.7759/cureus.91694

**Published:** 2025-09-05

**Authors:** Sudeep Saran, Avina Kharat, G Harsha Vardhan Reddy, Balakrishnan Madhavan, Aditya Bhattacharjee, Milan Paul Thomas

**Affiliations:** 1 Department of Diabetologist and Critical Care, Saran Hospital and Institute of Paramedical Science, Bareilly, IND; 2 Department of Pharmacology, ESIC (Employees State Insurance Corporation) Medical College and Hospital, Indore, IND; 3 Department of HPB (Hepato-Pancreato-Biliary) Surgery and Liver Transplantation, Institute of Liver and Biliary Sciences, New Delhi, IND; 4 Department of Ophthalmology, Sree Balaji Medical College and Hospital, Bharath Institute of Higher Education and Research, Chennai, IND; 5 Department of Medicine and Surgery, Amrita School of Medicine, Faridabad, IND; 6 Department of Oncology, Malankara Orthodox Syrian Church Medical College, Kolenchery, IND

**Keywords:** anaesthetic management, chronic kidney disease, perioperative care, pharmacokinetics, renal dysfunction

## Abstract

Chronic kidney disease (CKD) poses significant challenges for perioperative anaesthetic management due to complex alterations in drug clearance, fluid and electrolyte balance, and increased cardiovascular risk. Despite growing prevalence, there is a lack of consolidated clinical guidance addressing these issues from an internal medicine perspective. This review aims to comprehensively synthesize current evidence on anaesthetic considerations in CKD patients, focusing on strategies to optimize perioperative care. A thorough literature review was conducted, examining pathophysiological changes, pharmacokinetic alterations, and clinical management protocols relevant to this population. Key findings emphasize the necessity for meticulous preoperative assessment, including evaluation of renal and cardiovascular function and careful medication adjustment to avoid nephrotoxicity. Intraoperative management should prioritize individualised anaesthetic choice, vigilant fluid and hemodynamic monitoring, and prevention of acute kidney injury. Postoperative care involves renal function preservation, tailored analgesia, and infection prevention, with special attention to dialysis and transplant patients. The review underscores the critical role of an interdisciplinary approach integrating nephrology, anaesthesia, and internal medicine. Implementing these strategies can improve perioperative safety and reduce complications. Future research is needed to establish evidence-based guidelines tailored to this vulnerable population.

## Introduction and background

Chronic kidney disease (CKD) is a serious and increasingly important health problem in all parts of the world, and it is estimated that 10%-15% of the adult population is suffering from the disease in the entire world. The significant contributor to this increase is the escalating incidence of diabetes mellitus and hypertension, which are the two most prominent causes of CKD [1,2]. CKD is defined as the destruction or malfunction of the kidneys lasting for at least three months [3]. The disease progresses through five stages, the mild impairment (stage 1) progressing to the end-stage renal disease (ESRD), which needs either dialysis or transplantation (stage 5) [4]. Besides progression to kidney failure, CKD itself is a powerful independent risk factor of cardiovascular morbidity and mortality, which complicates the management of such patients [5].

Renal and non-renal surgical procedures are required in a large proportion of CKD patients. These patients have a great likelihood of experiencing perioperative complications, including cardiovascular events, bleeding complications, fluid and electrolyte imbalances, and acute kidney injury (AKI) [6,7]. The elevated risk is multifactorial and is provoked by the pathophysiological changes that involve the derangement of fluid and electrolyte homeostasis, immune dysfunction, anaemia, and cardiovascular remodelling [8]. Additionally, during CKD, the pharmacokinetic and pharmacodynamic changes occur, affecting the absorption, metabolism, and clearance of drugs, and result in the challenging management of anaesthetics [9]. To give an example, impaired renal clearance can lead to the build-up of anaesthetic drugs and their metabolites, creating a risk of toxicity. Hyperkalaemia and other electrolyte imbalances additionally put patients at risk of developing fatal arrhythmias intra-operatively [10].

**Figure 1:**
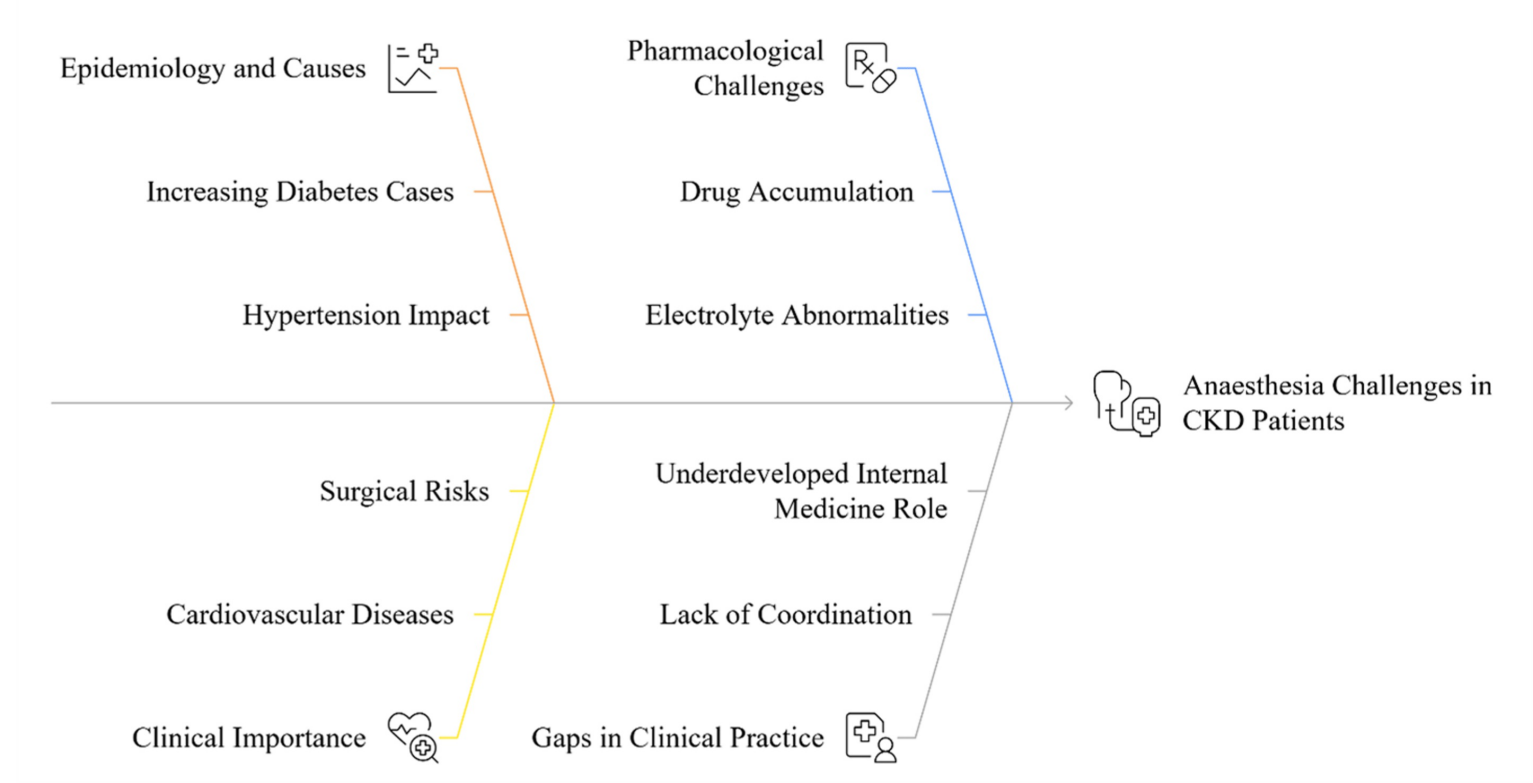
A Conceptual Framework for Managing Anaesthetic Challenges in Chronic Kidney Disease Image credit: Sudeep Saran. CKD: chronic kidney disease.

However, as the complexity and number of CKD patients undergoing surgery are increasing, the existing clinical guidelines commonly approach nephrology and anaesthesia as distinct issues, rather than consolidated and multidisciplinary. Internal medicine physicians have a key role in optimising comorbid conditions and medical treatments before surgery, but the relationship between internal medicine and anaesthesia in the perioperative management of CKD has not been well developed and may result in fragmented care and poor outcomes [1,2]. Moreover, the perioperative practices often fail to capture the finer details like customised changes in medication dosage, fluids, and dialysis timing according to the degree of kidney damage. Multi-disciplinary model including nephrology, internal medicine, and anaesthesia is needed to personalise care and minimise complications and improve surgical outcomes [3]. Figure 1 summarises the complex anaesthetic issues in chronic kidney disease and identifies several important topics including disease epidemiology, pathophysiology, pharmacological aspects, perioperative risks, clinical gaps, and the importance of multidisciplinary collaboration in providing optimum patient care.

Objectives of the review

The proposed review will synthesise the existing body of knowledge regarding anaesthetic management in patients with chronic kidney disease with the view of developing an effective perioperative care plan. In particular, it discusses the pathophysiological alterations and pharmacokinetic modifications that are typical of CKD and which affect the anaesthetic management. The review focuses on careful preoperative assessment, especially renal and cardiovascular assessment, and cautious modifications of medication to lessen nephrotoxicity. It also discusses personalised intraoperative anaesthetic strategies, close fluid and hemodynamic monitoring, and pre-emptive acute kidney injury measures. Renal function preservation, analgesia that is customised, and infection prevention are all considerations in postoperative care, and particular consideration is made to dialysis and transplant patients. In the end, this review highlights the utmost importance of an interdisciplinary approach combining nephrology, anaesthesia, and internal medicine in order to improve perioperative safety and decreased complications in this at-risk population.

## Review

Pathophysiological changes in CKD affecting anaesthesia

Chronic kidney disease (CKD) is one of the conditions whose features include the inability to regulate fluid and electrolyte homeostasis, which can have dire consequences in the course of anaesthetic management. Progression of renal impairment decreases the ability of the kidney to maintain sodium and water homeostasis, and consequently, patients tend to become volume overloaded or depleted depending on intake and losses and perioperative fluid administration. These changes are especially critical in the operating room, where their uncontrolled poor management can provoke pulmonary oedema or hypotension [11]. Electrolyte disturbances are frequent and clinically significant. Hyperkalaemia, which is caused by impaired renal excretion of potassium, is associated with a great risk of life-threatening cardiac arrhythmias during anaesthesia [12]. Intraoperative management is complicated by metabolic acidosis, which, because of its effect on acid excretion, alters drug ionization and metabolism and thus may influence drug activity [13]. Other imbalances like hypocalcaemia and hyperphosphatemia may interfere with neuromuscular integrity and cardiac stability, adding risk in the perioperative period [14].

These disturbances are closely connected with the most common cardiovascular changes in CKD. The disease itself is a powerful independent risk factor for cardiovascular pathology; numerous patients have hypertension, left ventricular hypertrophy, and diastolic dysfunction [4,15]. These structural and functional alterations compromise the performance of the myocardium and increase the susceptibility to ischemic episodes, arrhythmias, and heart failure in the operative period. Increased atherosclerosis and vascular calcification that frequently occur in CKD make the arteries stiff, which hinders coronary perfusion [16]. The autonomic dysfunction common in later CKD stages attenuates compensatory mechanisms to haemodynamic changes during anaesthesia, exacerbating hypotension and ischemia risks [17]. Volume overload and anaemia that occur concurrently elevate myocardial oxygen requirements, which places additional strain on the heart in the perioperative period [18].

One of the most frequent complications of CKD is anaemia, which is largely motivated by a decrease in the synthesis of erythropoietin. It reduces global oxygen delivery, which worsens tissue hypoxaemia during anaesthesia and augments cardiovascular risk [19]. Uraemic toxins also affect the functioning of platelets, leading to a bleeding diathesis in spite of normal platelet counts and making haemostasis difficult [20]. Changes in coagulation factors, as well as the common use of anticoagulants due to comorbid cardiovascular disease, increase the risks of bleeding. Therefore, preoperative correction of haematologic abnormalities is necessary to minimize perioperative blood loss and enhance outcomes.

**Figure 2:**
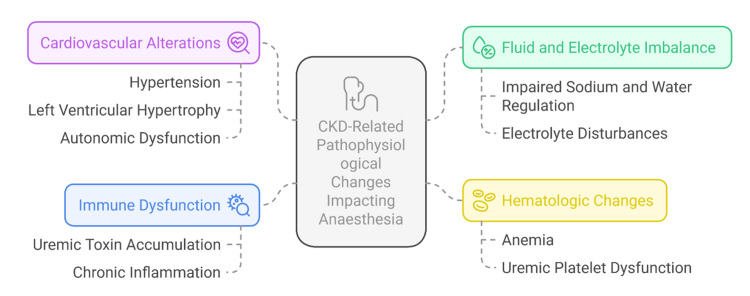
Pathophysiological Changes in Chronic Kidney Disease Affecting Anaesthetic Management Image credit: Sudeep Saran. CKD: chronic kidney disease.

Immune dysfunction is another significant concern. The build-up of uraemic toxins, chronic inflammation, and nutritional deficiencies weakens both innate and adaptive immunity of CKD patients [9]. This immunosuppression increases the risk of infection, such as at surgical sites and sepsis, which can be further aggravated in the case of dialysis or transplant patients. In this regard, thorough perioperative infection precautions and individualised prophylactic antibiotic plans are justified [11]. Figure 2 illustrates the major pathophysiological alterations in chronic kidney disease (CKD) that impact anaesthetic care.

Pharmacokinetic and pharmacodynamic alterations in CKD

Chronic kidney disease is known to profoundly change the pharmacokinetics and pharmacodynamics of many anaesthetic and adjunctive drugs, such that specific consideration in the perioperative period is required to prevent toxicity and achieve therapeutic outcomes [1]. Although gastrointestinal absorption is usually not impaired, uraemia-induced gastroparesis may slow down the absorption of some drugs [18]. More importantly, CKD alters drug distribution, metabolism, and excretion. Hypoalbuminemia, common in CKD, decreases plasma protein binding of acidic drugs, thus elevating the free active drug fraction and potentially causing toxicity [13]. The volume of distribution of hydrophilic drugs is increased by the increased extracellular fluid volume that is seen in uraemia, and often such drugs need an increased loading dose to reach therapeutic concentrations [4].

Secondary hepatic metabolic dysfunction can occur as a result of systemic effects of renal failure and metabolite build-up. Phase I (oxidation, reduction) reactions are more influenced than Phase II conjugation reactions (glucuronidation, sulfation) that are usually preserved [15]. The most affected elimination route, however, is renal excretion, leading to accumulation of renally cleared drugs and active metabolites, which extend drug half-life and risk of adverse effects [16]. Clinically, these changes impact commonly used anaesthetic agents. Active metabolites of morphine build up in CKD and pose a risk of enhanced sedation and respiratory depression, so dose modifications or substitutes are needed [7]. Fentanyl and remifentanil, which are not dependent on renal clearance, are favoured regarding safety [10]. Vecuronium and rocuronium have long duration in CKD as their clearance is decreased, and atracurium and cisatracurium, which are metabolised through Hofmann degradation, are better choices [19]. Benzodiazepines build up active metabolites in renal failure, increasing the risk of sedation and delirium, and propofol clearance is not significantly altered but requires careful cardiovascular observation [20]. Volatile anaesthetics are mostly safe, with minimal renal metabolism, although close cardiovascular monitoring is indicated due to the common CKD-related cardiac dysfunction [13].

Preoperative assessment in CKD patients

A thorough preoperative assessment is crucial to reducing perioperative morbidity in patients with chronic kidney disease (CKD). Beyond relying solely on serum creatinine, more accurate renal function evaluation using estimated glomerular filtration rate (eGFR) or creatinine clearance is necessary for appropriate staging and risk stratification [4]. Correction of electrolyte imbalances such as hyperkalaemia, hyponatremia, and metabolic acidosis before surgery is vital to prevent cardiac and metabolic complications [12]. Further assessment through proteinuria measurement and dialysis adequacy evaluation helps define disease severity and systemic effects, guiding perioperative planning [9,12]. Given the high cardiovascular risk, comprehensive cardiac evaluation, including history, physical examination, electrocardiography, and echocardiography, and optimization of comorbidities like anaemia, hypertension, and diabetes are essential to minimise perioperative events [14,16]. Careful review of medication regimens to avoid nephrotoxic drugs and adjust dosing based on renal clearance reduces toxicity risks [17]. Effective collaboration among nephrology, internal medicine, anaesthesia, and dialysis teams is critical to implementing individualised and safe perioperative care plans [1]. Monitoring and preserving vascular access integrity in dialysis-dependent patients further helps prevent procedural complications [19]. The following table summarises these critical assessment and management components along with their clinical objectives. Table 1 summarises the essential elements of preoperative evaluation and management for patients with chronic kidney disease undergoing surgery.

**Table 1:** Key Components of Preoperative Assessment and Optimization in Chronic Kidney Disease Patients eGFR: estimated glomerular filtration rate, CKD: chronic kidney disease.

Assessment Area	Key Actions	Purpose/Outcome	References
Renal function evaluation	Use eGFR or creatinine clearance in addition to serum creatinine	Accurate CKD staging and risk stratification	Craig and Hunter, 2008 [4]
Electrolyte correction	Correct hyperkalaemia, hyponatremia, metabolic acidosis	Reduce perioperative cardiac and metabolic risks	Keith et al., 2004 [12]
Additional renal tests	Measure proteinuria and acid-base balance	Assess disease severity and systemic impact	Keith et al., 2004 [12]
Dialysis coordination	Assess dialysis adequacy and schedule perioperative dialysis	Optimize fluid and electrolyte balance	Shazad et al., 2023 [9]
Cardiovascular assessment	Conduct history, physical exam, ECG, echocardiography; consider stress testing or angiography if indicated	Identify ischemia, heart failure; stratify risk	Plantinga et al., 2008 [14]
Hemodynamic optimization	Optimize blood pressure and volume status	Decrease perioperative cardiovascular events	Strøm and Rasmussen, 2014 [16]
Anaemia management	Correct anaemia preoperatively	Improve oxygen delivery and reduce complications	Einhorn et al., 2009 [13]
Comorbidity management	Optimize diabetes, hypertension, and peripheral vascular disease	Minimize perioperative risk factors	Rang et al., 2006 [6]
Medication review	Avoid nephrotoxic drugs; adjust doses for renal clearance	Prevent nephrotoxicity and drug toxicity	Kehlet and Dahl, 2003 [17]
Vascular access monitoring	Track and preserve dialysis access integrity	Prevent access-related complications	Sear, 2005 [11]
Team coordination	Collaborate among nephrology, internal medicine, anaesthesia, and dialysis teams	Ensure integrated, safe perioperative care	Jaszczuk et al., 2022 [7]

Medication management and nephrotoxicity prevention

Optimal perioperative medication management is central in reducing nephrotoxicity and enhancing outcomes in patients with chronic kidney disease (CKD). Known nephrotoxic agents should be avoided or doses altered by the clinicians. Nonsteroidal anti-inflammatory drugs (NSAIDs), which are frequently utilised in analgesia, diminish the production of renal prostaglandins, resulting in the reduction of renal blood flow, and thus, may trigger the acute kidney injury (AKI), especially in volume-depleted or hemodynamically unstable patients [21]. Aminoglycoside-based antibiotics, which are effective in gram-negative infections, accumulate in renal tubular cells and cause direct toxicity, and hence, their dosing should be individualised according to renal function with therapeutic drug monitoring to avoid injury [22].

Anticoagulants and antiplatelet agents also represent a challenge since they have changed pharmacokinetics and increase the risk of bleeding in CKD. Warfarin has variable protein binding and drug interactions, which influence its metabolism, and this requires frequent monitoring of the international normalised ratio (INR). The novel oral anticoagulants (NOACs) differ in their levels of renal clearance, and their doses should be adjusted or avoided based on kidney function [23]. Although antiplatelet agents such as clopidogrel are minimally cleared renally, perioperative timing is of great importance in balancing the risks of bleeding and thrombosis [24]. Close collaboration of nephrologists, haematologists, and anaesthesiologists in a multidisciplinary manner is required to design personalised anticoagulation plans.

The essential part of nephrotoxicity prevention is the optimization of volume status before surgery. Hypovolemia decreases renal perfusion and increases the risk of AKI, and fluid overload worsens hypertension and cardiac strain. Clinical assessment of preoperative euvolemia, with the addition of hemodynamic monitoring when possible, should be targeted to the needs of the individual [25]. This is particularly important in dialysis patients, where dry weight and interdialytic weight gains are used to guide fluid management to prevent perioperative complications.

Intraoperative anaesthetic management

The intraoperative care of CKD patients needs a personalised anaesthetic strategy taking into consideration the needs of the surgery, comorbidities, and the effect on renal function. Where possible, regional anaesthesia is preferred because it provides limited systemic drug exposure, enhances analgesia, and blunts the sympathetic stress response, which may preserve renal perfusion [26]. Neuraxial techniques can, however, be contraindicated in the presence of coagulopathy or in cases where the patient has received anticoagulant therapy in the recent past, and a careful assessment of risks is necessary. In the case of general anaesthesia, where renal excretion and inactive metabolites are drugs of choice, fentanyl and cisatracurium will be preferred to avoid long sedation and toxicity [27]. Propofol, a common induction agent, should have close attention paid to blood pressure as it has vasodilatory properties which can result in hypotension and compromised renal perfusion. Volatile anaesthetics are otherwise safe except that they are to be used with caution in patients who are cardiovascularly compromised.

**Figure 3:**
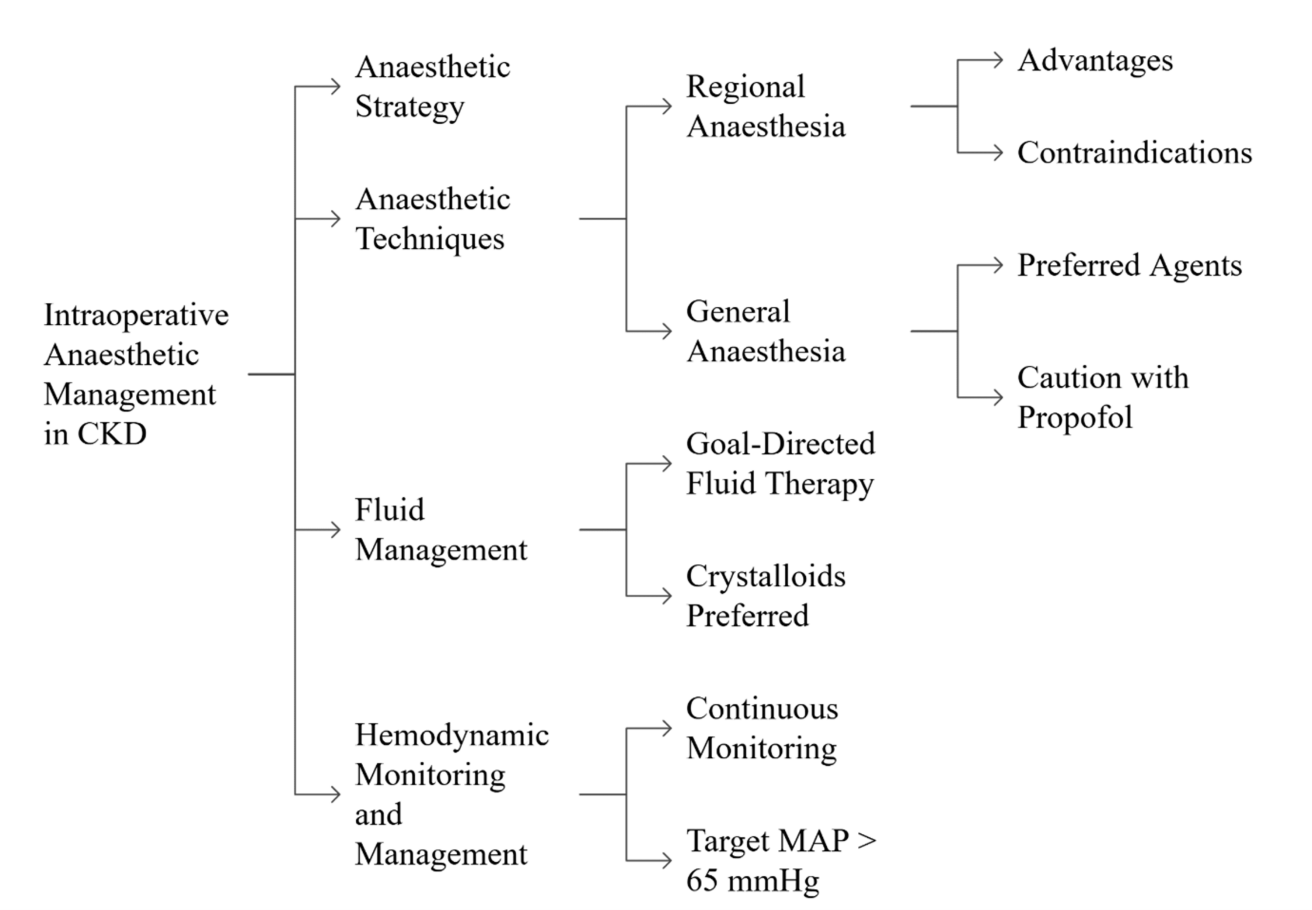
Structured Overview of Intraoperative Anaesthetic Management in Chronic Kidney Disease Image credit: Sudeep Saran. CKD: chronic kidney disease, MAP: mean arterial pressure.

Intraoperative fluid management aims to maintain optimal fluid status that would not compromise renal function but would prevent fluid overload. Dynamic hemodynamic goal-directed fluid therapy has shown better results than fixed-volume fluid administration in that it personalises fluid administration and decreases postoperative complications [28]. The preference for crystalloids over synthetic colloids is based on the issue of colloid-related renal toxicity. Despite the fact that urine production is a classic parameter of renal functional activity, it is necessary to consider it in combination with mean arterial pressure (MAP) and cardiac output to manage fluids and vasopressors most effectively. Uninterrupted hemodynamic observation allows identifying and treating hypotension early, which is one of the most important modifiable risks of AKI. It is advised to keep MAP above 65 mmHg, and the goals should be personalised, depending on the baseline blood pressure and comorbidities [29]. More advanced monitoring methods, such as arterial waveform analysis and cardiac output measurement, can help guide fluid resuscitation and vasopressor administration. Combined, hemodynamic stability and the avoidance of nephrotoxins are most important to safeguard renal function during surgery. Figure 3 summarises intraoperative anaesthetic management in CKD, highlighting personalised strategies, benefits, and contraindications of regional anaesthesia, and preferred agents with cautions for general anaesthesia.

Prevention and management of acute kidney injury (AKI)

AKI is a frequent and severe perioperative complication in CKD patients, which greatly enhances morbidity and mortality. Early identification of risk factors facilitates targeted prevention. Intraoperative factors involve long-standing hypotension, exposure to nephrotoxic drugs, massive blood loss, and systemic diseases that result in renal hypoperfusion, like sepsis or heart failure [30]. Thorough preoperative risk stratification that includes comorbidities, baseline renal function, and complexity of surgery can help to guide individual preventive approaches. The prevention of AKI is based on the maintenance of sufficient renal perfusion and oxygen delivery throughout surgery. Ischemic tubular injury is reduced by optimization of hemodynamics using fluid resuscitation and vasoactive support. The most important thing is to avoid prolonged hypotension and ensure adequate cardiac output [1]. Other protective measures are limiting the amount of aortic cross-clamping time used during vascular operations and avoiding rhabdomyolysis during long operations.

New biomarkers Neutrophil gelatinase-associated lipocalin (NGAL), kidney injury molecule-1 (KIM-1), and cystatin C hold the promise of detecting tubular damage earlier than increases in serum creatinine. Although these biomarkers are yet to be used in a clinical setting extensively, they hold the promise of timely intervention and improved prognosis [21]. The treatment of known AKI focuses on supportive care, avoidance of additional nephrotoxins, and fluid and electrolyte homeostasis. Severe cases that have refractory volume overload, electrolyte imbalance, or uraemia are treated using renal replacement therapy. The use of multidisciplinary coordination leads to the start of dialysis in time and full control of complications [22]. Fluid management bundles that include protocols to avoid nephrotoxins and hemodynamic optimization have also shown a reduction in the incidence and severity of AKI in high-risk surgical patients [23].

Postoperative care and monitoring

Postoperative care in patients with chronic kidney disease (CKD) demands vigilant monitoring to prevent complications and promote renal recovery. Baseline laboratory tests, including serum creatinine, blood urea nitrogen, and electrolytes, are essential for early detection of abnormalities such as hyperkalaemia and metabolic acidosis, enabling timely interventions [31]. Pain management must account for altered drug handling in CKD, favouring opioid dose reductions and safer agents like fentanyl and hydromorphone, while avoiding nephrotoxic nonsteroidal anti-inflammatory drugs (NSAIDs) [32,33]. Infection prevention is critical due to immunosuppression; appropriate dosing of prophylactic antibiotics and stringent aseptic techniques reduce infection risks [34]. Fluid management requires a delicate balance to maintain renal perfusion without causing overload or depletion. Individualised therapy guided by clinical and hemodynamic parameters optimises outcomes [35]. For dialysis-dependent patients, coordination with nephrology regarding dialysis timing and access care is vital to prevent complications [36]. Table 2 summarises the essential elements of postoperative management for patients with chronic kidney disease undergoing surgery.

**Table 2:** Key Components of Postoperative Care in Patients With Chronic Kidney Disease NSAIDs: nonsteroidal anti-inflammatory drugs.

Care Component	Key Actions	Clinical Goals	References
Laboratory monitoring	Measure serum creatinine, blood urea nitrogen, and electrolytes	Early detection of renal and metabolic derangements	Yaxley, 2025 [31]
Pain management	Use reduced opioid doses; prefer fentanyl/hydromorphone; avoid NSAIDs	Prevent drug accumulation and nephrotoxicity	Baker et al., 2002 [32], Rajakumar et al., 2016 [33]
Infection prevention	Administer renal-dose prophylactic antibiotics; ensure aseptic technique	Minimize surgical site infections and sepsis	Shaheen et al., 2018 [34]
Fluid management	Employ personalised fluid therapy guided by clinical and hemodynamic data	Maintain renal perfusion; avoid hypovolemia/overload	Fiekabo and Victor, 2023 [35]
Dialysis coordination	Collaborate with nephrology on dialysis timing and vascular access care	Optimize volume/electrolyte balance; prevent access complications	Deo et al., 2023 [36]

Anaesthesia considerations in dialysis patients

Dialysis patients have unique perioperative issues that pose specific challenges that need careful planning and multidisciplinary coordination. The timing of dialysis in reference to surgery is very crucial in perioperative stability. Elective operations should be planned on the day following dialysis in order to maximize fluid status, electrolyte abnormalities, and metabolic disturbances before the administration of anaesthesia [37]. Thorough preoperative assessment and close intraoperative observation are mandatory in emergencies. Dialysis causes significant fluid and electrolyte shifts perioperatively. The loss of the excess fluid and solutes can lead to intravascular volume contraction, which predisposes the occurrence of intraoperative hypotension and renal hypoperfusion [38]. On the other hand, an interdialytic weight gain indicates the presence of fluid overload, and it predisposes to the development of pulmonary oedema and hypertension when it is not addressed effectively. Close monitoring of electrolyte imbalances, particularly of potassium and calcium, is required to avoid arrhythmias and neuromuscular dysfunction. These dynamic changes should be predicted in the anaesthetic plans to ensure hemodynamic stability and electrolyte balance.

Anticoagulation management in dialysis patients requires special attention. Heparin, which is regularly administered during haemodialysis to avoid clotting of the circuit, poses a risk of bleeding when surgery is performed in close succession. Nephrology and surgical teams should coordinate to make adjustments to anticoagulation, which may include using heparin-free dialysis or changing the timing of administration [39]. The integrity of vascular access sites must be preserved; intravenous lines and blood pressure cuffs must not be used on the limbs with arteriovenous fistulas or grafts to avoid thrombosis or damage. Vascular access can be evaluated preoperatively to aid in planning and reducing complications to provide successful postoperative dialysis [40].

Anaesthetic challenges in kidney transplant recipients

Kidney transplant recipients present distinct anaesthetic challenges due to their immunosuppressed state, heightened infection risk, cardiovascular comorbidities, and complex pharmacotherapy [41]. Immunosuppressive agents such as calcineurin inhibitors (tacrolimus, cyclosporine), antimetabolites (mycophenolate mofetil), and corticosteroids substantially weaken the immune system, necessitating increased perioperative vigilance and strict aseptic methods [42]. Cardiovascular disease remains the leading cause of morbidity and mortality in this population, with common conditions like hypertension, left ventricular hypertrophy, and ischemic heart disease often worsened by chronic immunosuppression and metabolic derangements such as dyslipidaemia and diabetes mellitus [43]. Maintaining graft function requires close monitoring of renal parameters, including urine output, serum creatinine, and hemodynamics, alongside anaesthetic techniques focused on preserving renal perfusion and avoiding hypotension and nephrotoxic drugs [44]. Metabolism of the calcineurin inhibitors by the cytochrome P450 system poses the risk of drug interactions with numerous perioperative drugs, such as antibiotics, antifungals, and sedatives, which could result in toxicity or rejection [45]. This intricacy requires tight coordination with transplant pharmacologists in making drug alterations. There is also a possibility of neuromuscular blocker effects to be enhanced by altered drug metabolism and compromised renal clearance, which requires close dosing and neuromuscular monitoring [46]. Table 3 provides a summary of the important anaesthetic issues specific to kidney transplant recipients; these are immunosuppression, cardiovascular comorbidities, drug interactions, and neuromuscular blocker issues.

**Table 3:** Anaesthetic Challenges and Management Considerations in Kidney Transplant Recipients

Challenge	Details	Management Considerations	References
Immunosuppression and infection risk	Use of calcineurin inhibitors (tacrolimus, cyclosporine), antimetabolites (mycophenolate mofetil), and corticosteroids leading to immune suppression	Increased vigilance, strict aseptic technique, and customised prophylactic antibiotics	Abdalla et al., 2025 [23]
Cardiovascular comorbidities	Hypertension, left ventricular hypertrophy, ischemic heart disease worsened by immunosuppression and metabolic issues (dyslipidaemia, diabetes mellitus)	Monitor renal function and hemodynamics closely; avoid hypotension and nephrotoxins	Shaheen et al., 2018 [34]
Drug interactions	Calcineurin inhibitors are metabolised by cytochrome P450; interactions with antibiotics, antifungals, and sedatives	Collaborate closely with transplant pharmacologists; adjust drug regimens	Meredith et al., 2021 [45]
Neuromuscular blocker effects	Altered metabolism and impaired renal clearance prolong neuromuscular blockade	Dose adjustment and neuromuscular monitoring are required	Strøm and Rasmussen, 2014 [16]

Role of the interdisciplinary team in perioperative care

The complexity of issues in the care of CKD patients, who are also transplant patients, requires an interdisciplinary team effort comprising nephrologists, anaesthesiologists, surgeons, and internists. This type of collaboration can guarantee the thorough assessment of patient-specific risks and support the creation of customised perioperative care plans to meet the complexity of CKD and its comorbidities [47]. The involvement of all specialties as early as possible facilitates the best preoperative preparation, intraoperative management, and postoperative follow-up and decreases the number of complications, and improves the outcomes.

The heterogeneity of clinical presentations of the CKD population necessitates individualised care. A collective evaluation of the cardiovascular status, renal reserve, medication profiles, and psychosocial factors by the interdisciplinary team will help in the perioperative strategies tailoring process. Such risk reduction strategies as control of blood pressure, fluid balance, immunosuppressive protocols adjustment, and dialysis planning need to be included in patient-specific plans [48]. Such a person-centred practice integrates treatment goals and patient values, enhancing safety and satisfaction. Successful interdisciplinary care hinges on effective communication and collaborative decision-making [49]. Multidisciplinary meetings are held regularly, and the utilization of standardised communication tools, including checklists and integrated electronic health records, helps to exchange information, reduce errors, and promote an agreement on perioperative management [50]. Engaging patients and their families in risk, benefit, and expectation discussions also increases adherence and satisfaction. Finally, this teamwork-based, patient-centred approach is the solution to managing the multifaceted needs of CKD patients undergoing surgical interventions and enhancing perioperative outcomes [51].

Methodological considerations and limitations

The review was performed using a systematic literature search in essential biomedical databases such as PubMed, Embase, and the Cochrane Library. We used specific keywords and Medical Subject Headings (MeSH) terms, including “chronic kidney disease,” “anaesthesia,” “perioperative management,” “pharmacokinetics,” and “acute kidney injury.” The literature search was limited to the English language and publications over the past two decades. We aimed to obtain a broad range of evidence covering the anaesthetic management of CKD patients undergoing surgery; thus, we did not limit the study designs and included randomised controlled trials, prospective cohort studies, and retrospective studies.

In spite of this stringent methodology, there was considerable heterogeneity among studies that presented challenges to data synthesis. The literature included was highly heterogeneous concerning methodology and patient populations, with small case series, retrospective cohorts, and only a few prospective trials. Such inconsistency makes it more difficult to combine results and limit the development of absolute, evidence-based clinical practice guidelines. Also, there are very few randomised controlled trials specifically addressing anaesthetic concerns in CKD patients, which reduces the level of existing recommendations [52]. Differences in CKD stages, comorbidities, and types of surgery additionally limit the external validity of findings. In addition, the lack of consistency in the definition of the severity of renal impairment and perioperative outcomes, including AKI incidence, cardiovascular events, and mortality, prevents consistent interpretation. These methodological shortcomings highlight the dire necessity of properly designed prospective studies and reporting models to further inform perioperative anaesthetic management in CKD.

Future directions

Well-conducted randomised controlled trials to specifically optimise perioperative anaesthetic protocols in CKD patients need to be the focus of future studies. This is a major gap in evidence that should be addressed in order to produce standardised, multidisciplinary clinical guidelines with the capacity to transform patient outcomes. A paradigm shift in clinical decision-making will be achieved by innovations in new biomarkers that will have the capability to detect acute kidney injury (AKI) earlier and more accurately to enable timely interventions. There is also exploration of using pharmacogenomic approaches, which hold the potential of personalising drug dosing and thus reducing toxicity and improving therapeutic efficacy in this pharmacokinetically complex group of individuals. In addition, adapted enhanced recovery after surgery (ERAS) protocols to meet the particular physiological requirements of CKD patients may contribute to a reduction in perioperative complications and acceleration of the recovery. Collectively, these research domains are important to the evolution of perioperative care and improving safety in this at-risk and growing patient population.

## Conclusions

This is a review that incorporates the finest obtainable evidence with the intention of illuminating the intricate issues of chronic kidney disease (CKD) in perioperative anaesthetic management. It emphasises a cautious, patient-centred approach of meticulous preoperative investigation, personalised cooperative intraoperative anaesthetic technique, and close postoperative monitoring. This review addresses gaps in clinical synchrony, pharmacokinetic understanding, and perioperative practices in nephrology, internal medicine, and anaesthesia by reporting altered pharmacokinetics, risks with nephrotoxic drugs, and prevention of acute renal failure. It offers practical recommendations, including personalised drug dosing and fluid administration, which aim at decreasing perioperative damage and preserving renal function.

The synthesis of multidisciplinary perspectives is the unique feature of the review as it forms a coherent framework interconnecting various specialties to improve perioperative management of CKD patients. It highlights the key evidence gaps, such as the unavailability of randomised controlled trials and the unavailability of standardised guidelines, and strongly advocates targeted research as well as the development of multidisciplinary clinical guidelines. Besides that, the review highlights the prospects of new technologies, including new biomarkers and pharmacogenomics, or the use of enhanced recovery after surgery (ERAS) protocols in the CKD population. Finally, the study will be used to spur innovation in perioperative care, further enhancing safety and outcomes in this vulnerable and increasingly numerous patient group.
